# An ecological resilience model for adolescents with type 1 diabetes: a cross-sectional study

**DOI:** 10.1186/s12888-024-05634-1

**Published:** 2024-04-09

**Authors:** Dan Luo, Xue Cai, Hong Wang, Jingjing Xu, Yubing Wang, Mingzi Li

**Affiliations:** 1 School of Nursing, Nanjing University of Chinese Medicine, Jiangsu, China; 2 Department of Respiratory, Department of Nursing, School of Medicine, Zhongda Hospital, Southeast University, Jiangsu, China; 3 Department of Endocrinology, The First Affiliated Hospital of Nanjing Medical University, Jiangsu, China; 4 Department of Nursing, The First Affiliated Hospital of Nanjing Medical University, Jiangsu, China; 5 Department of Endocrinology, Children’s Hospital of Nanjing Medical University, Jiangsu, China; 6 School of Nursing, Peking University, Beijing, China

**Keywords:** Diabetes type 1, Adolescents, Resilience, Ecological determinants

## Abstract

**Background:**

Highly resilient adolescents with type 1 diabetes have been proved to achieve within-target glycemic outcomes and experience high quality of life. The ecological resilience model for adolescents with type 1 diabetes was developed in this study. It aims to increase our understanding of how resilience is both positively and negatively affected by internal and environmental ecological factors.

**Methods:**

This cross-sectional study surveyed 460 adolescents with type 1 diabetes from 36 cities in 11 provinces, China. Participants completed self-report questionnaires on resilience, family functioning, peer support, peer stress, coping style, and demographics. Standard glycated hemoglobin tests were performed on the adolescents. Structural equation modeling was applied to analyze the data.

**Results:**

The ecological resilience model for adolescents with type 1 diabetes was a good model with a high level of variance in resilience (62%). Family functioning was the most important predictor of resilience, followed by peer support, positive coping, and peer stress. Moreover, positive coping was the mediator of the relationship between family functioning and resilience. Positive coping and peer stress co-mediated the association between peer support and resilience.

**Conclusions:**

Family functioning, peer relationships, and positive coping are interrelated, which may jointly influence resilience. The findings provide a theoretical basis for developing resilience-promotion interventions for adolescents with type 1 diabetes, which may lead to health improvements during a vulnerable developmental period.

## Background

Type 1 Diabetes (T1D) is one of the most common pediatric medical illnesses. Due to rapid developmental, physiological, and hormonal changes of puberty, adolescents with T1D are challenged to cope with their daily self-management tasks [1]. Over the past 15 years, around 76.4% of children and adolescents with T1D globally failed to meet the glycemic targets (glycosylated hemoglobin, HbA_1c_ < 7.5%) [2]. Moreover, adolescents with T1D are at increased risk for psychosocial concerns, including increased symptoms of depression, anxiety, distress, and diminished quality of life [3]. Despite the challenges of living with T1D, some adolescents achieve good psychosocial and health outcomes. Resilience is a quantifiable and modifiable personal capacity that enables individuals to overcome adversity and maintain well-being [4]. Available studies have shown that higher levels of resilience were positively associated with lower HbA_1c_, less diabetes distress, better self-management and improved quality of life [5–7]. The mounting empirical attention to the positive relationship between resilience and health outcomes among this population is encouraging. However, less is known about factors that influence adolescents’ resilience. Identifying contributing factors to resilience can help to develop resilience-promotion interventions, which may lead to health improvements in adolescents with T1D.

## Framework

According to the Ungar’s Social Ecology of Resilience Framework [8], resilience is characterized in terms of both internal and environmental ecological factors. Common internal factors include positive coping styles, cognitive ability, and emotional competence. Environmental factors include the microsystem (e.g., family, peers, and teachers), mesosystem (interactions between microsystems), exosystems, and macrosystem. Specifically, microsystem interactions determine the nature of the supportive resources available to individuals, influencing their resilience. Ecologically speaking, the interplay and interaction between internal and environmental ecological factors result in varying levels of resilience.

The Social Ecology of Resilience Framework is believed to best capture the individualized process of adjusting to stress, but does not specifically address T1D. Therefore, the current study examines the effects of internal ecological factors, microsystem environmental factors, and their interactions on the resilience of adolescents with T1D. We selected ecological factors from the Social Ecology of Resilience Framework and chose coping style as one of the internal ecological factors that affect adolescents’ resilience. Defined as the process of managing demands that are appraised as taxing or exceeding personal resources [9], coping is considered essential for resilience among people with chronic disease [10, 11]. Furthermore, we chose family functioning, peer support, and peer stress to represent microsystem environmental ecological factors since adolescents spend a sizable portion of their days in family and school. Family functioning refers to the emotional adhesion between family members and the ability of the family to respond with flexibility to various challenges [12]. A wide range of studies has shown a positive link between better family functioning and higher levels of resilience in patients with chronic disease [13, 14]. Peer support means instrumental, emotional, or informational resources from friends or schoolmates [15]. Peer stress includes friends’ or schoolmates’ negative thoughts and reactions about diabetes [16]. The peer-support approach proved helpful for enhancing resilience in different populations [17, 18]. In the case of adolescents with T1D, relatively little research has studied the association between peer relationships and resilience. Moreover, no study has investigated relations among family functioning and resilience in the context of peer factors.

Existing literature have explored the interrelationships between the above factors. A study of cancer couples has shown that family functioning was associated with resilience through peer support [19]. Ye et al. reported that positive coping mediated the association between peer support and resilience in parents of children with cancer [20]. Although family functioning and individual coping style have been demonstrated to be associated with resilience among adolescents with T1D [21], no studies have explored the interrelation association between family functioning, peer relationships, coping style, and resilience.

**Fig. 1:**
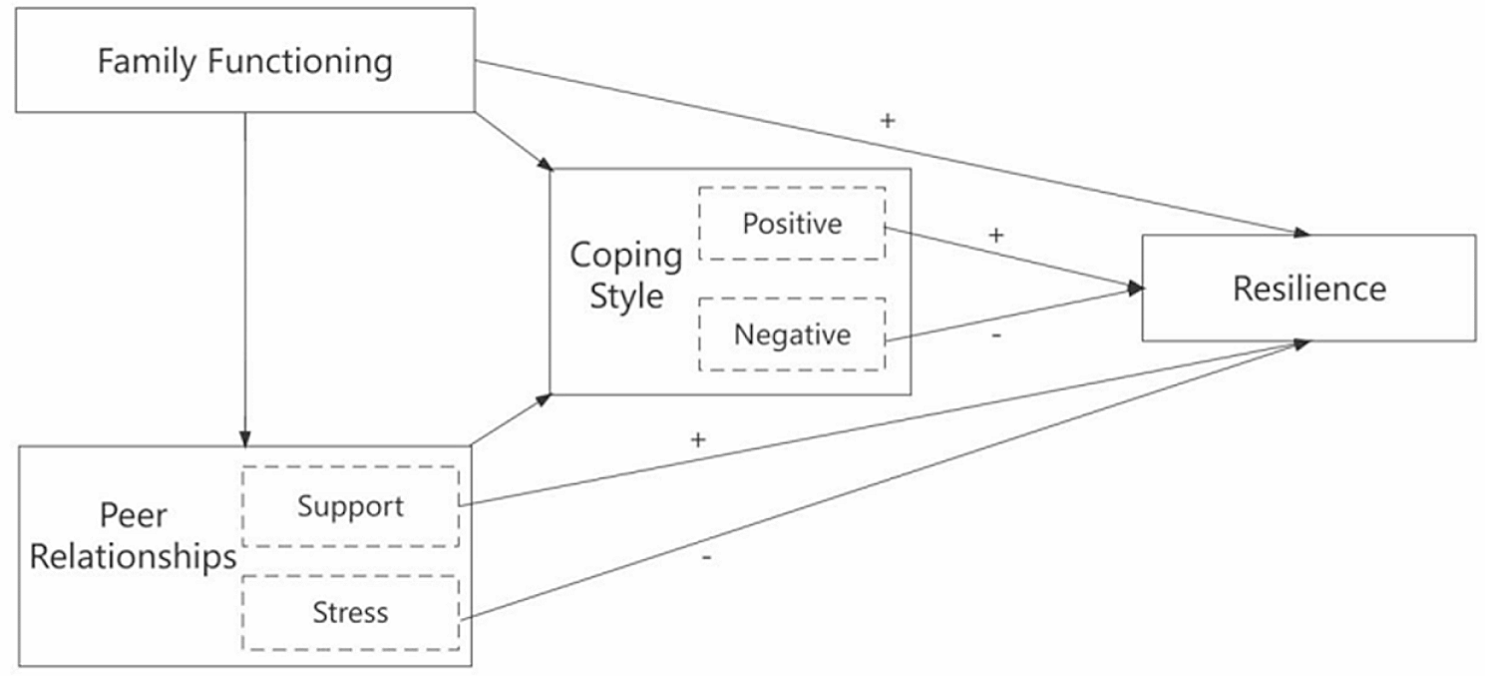
Hypothesized ecological resilience model for adolescents with T1D

This study employed the ‘Social Ecology of Resilience Framework’ as a guide to test an ecological resilience model for adolescents with T1D, thus laying the groundwork for developing targeted clinical interventions explicitly aimed at enhancing resilience. In line with Social Ecology of Resilience Framework, we proposed four main hypotheses: (1) family functioning, peer support, and positive coping were directly positively associated with resilience; (2) peer stress and negative coping were directly negatively associated with resilience; (3) peer relationships and coping style mediated the link between family functioning and resilience; (4) coping style mediated the link between peer relationships and resilience. The hypothesized ecological resilience model are delineated in Fig. 1.

## Methods

### Design and sample

This cross-sectional survey was conducted from June 2019 to October 2021. A convenience sample of participants was recruited from two endocrinology units in two academic hospitals in Jiangsu, China. Inclusion criteria were: (i) diagnosed with T1D for more than six months, (ii) age between 10 and 19 years, (iii) fluent in oral Mandarin, and (iv) able to understand and complete the questionnaire. Participants were excluded if they were (i) suffering from major psychiatric disorders (e.g., schizophrenia, major depressive disorder, bipolar disorder, substance use disorder) or neurocognitive disorder (e.g., delirium and dementia), (ii) suffering from diabetic acute complications (e.g., diabetic ketoacidosis and hypertonic coma) within 3months, or (iii) not willing to participate in the study. The ratio of 10 to 15 responses for each unlabeled parameter is sufficient for testing structural equation modeling [22]. Given 31 unlabeled parameters in our hypothesized model, 434 (10*14) participants were the sufficient sample size.

A total of 634 adolescents with T1D visited the endocrine clinic of two hospitals during the study period. A research nurse contacted all adolescents. Of the 576 adolescents interested in participating and screened for eligibility, 482 were eligible. As 22 adolescents submitted incomplete questionnaires, data from the remaining 460 participants were ultimately used for the statistical analysis.

### Procedures

One well-trained researcher recruited participants from two hospitals and collected the data. Adolescents underwent structured pen-and-paper surveys in a meeting room without the presence of their guardians. The self-reported questionnaires included six parts: resilience, family functioning, peer support, peer stress, coping style, and demographics. The researcher was available to answer adolescents’ questions. On average, the entire survey took 30–35 min to finish. One diabetes specialist nurse completed the blood sampling. Capillary blood samples were collected from adolescents through finger sticks. HbA_1c_ was measured at a central laboratory shared by both recruitment sites utilizing the Afinion AS100 Analyzer (Orlando, Florida).

### Measurements

#### Demographics

Demographic and disease-related characteristics included age, gender, region of residence, only-child or not, educational level of primary caregiver, family monthly income, disease duration, age of onset, and insulin regimen.

#### Resilience

We measured resilience using the Diabetes Strengths and Resilience Measure for adolescents (DSTAR-Teen) [23]. Our team translated the DSTAR‐Teen into Chinese and proved its psychometric properties [24]. Twelve items measured two dimensions: diabetes-related confidence and help with diabetes management, on a 5-point Likert scale (1 = never, 5 = almost always). The total score ranged from 12 to 60, with a higher score indicating higher levels of resilience. The Cronbach’s α in this sample was 0.87.

#### Family functioning

The Chinese version of the Family Adaptability and Cohesion Evaluation Scales II (FACES II) was used to measure family functioning [25]. The scale contained 30 items in two dimensions: adaptability and cohesion. Items were rated on a 5-point Likert scale ranging from 1 (almost never) to 5 (almost always). The overall scores ranged from 42 to 162, with a higher score indicating better family functioning. The Chinese version of the FACES II has good validity and reliability [25]. The Cronbach’s α in this sample was 0.93.

#### Peer support

Peer support was assessed by the 6-item Diabetes-Specific Peer Support Measure (DSPSM) [15]. The emotional support dimension, which contained items 1 to 3, measured perceived understanding and encouragement about diabetes from friends. Adolescents rated each item for the frequency (1 = never, 5 = at least once a day). Items 4 to 6 belonged to the behavioral support dimension, assessing friends’ awareness of patients’ diabetes diagnosis and knowledge of diabetes management on a 5-point Likert scale (1 = strongly disagree, 5 = strongly agree). We combined six items to form a single diabetes-specific peer support score. The reported reliability and validity of the DSPSM were good [15]. The Chinese versions of DSPSM in this sample had acceptable internal consistency (Cronbach’s α = 0.86).

#### Peer stress

The peer stress subscale of the Diabetes Stress Questionnaire for Youths (DSQY) was used to measure peer-related diabetes stress [16]. The peer stress subscale comprised eight items rated on a 4-point Likert scale (0 = not at all, 3 = very much). The overall score ranges from 0 to 24, with higher scores indicating more peer stress. The DSQY has been used in different populations with good psychometric properties [26]. The Cronbach’s α of the peer stress subscale was 0.92 in this sample.

#### Coping style

Coping style as assessed with the 20-item Simplified Coping Style Questionnaire (SCSQ) [27]. The SCSQ was developed for Chinese adolescents and measured three aspects of coping: self-regulation, fantasy and escape, help-seeking and problem-solving. Each item was rated from 0 (not used) to 3 (always using). The overall scores for self-regulation and help-seeking and problem-solving dimensions ranged from 0 to 39, with a higher score indicating a more positive coping style. The fantasy and escape dimension score ranged from 0 to 21, with a higher score indicating a more negative coping style. The SCSQ showed good test-retest reliability and construct validity [27]. In this sample, the Cronbach’s α of the positive and negative coping dimensions were 0.89 and 0.77, respectively.

### Data analysis

Data analyses were performed using IBM SPSS (version 22.0; SPSS Inc.) and AMOS (version 25.0; Amos Development Corporation). Descriptive statistics in the form of means with standard deviations (SD) or frequencies with percentages are displayed to describe the respondent’s characteristics. We used logistic regression to select demographics for the multivariate analyses to predict resilience. Adolescents were empirically classified into high (scoring in the highest 27%) and low resilience groups (scoring in the lowest 27%) according to their DSTAR-Teen score. For the unranked variables, dummy variables were created, such as gender, region of residence, and insulin regimen, which were then entered into multivariate analyses. The Pearson correlation coefficients (*r*) were calculated to evaluate the un-adjusted associations between the main study variables. The structural equation modeling (SEM) with a maximum likelihood estimation method was used to test the hypothesized ecological resilience model (Fig. 1). we subjected the model to confirmatory factor analysis (CFA) to affirm the measurement variable for the latent variables. Paths with non-significant coefficients were removed from the model. A bias-corrected bootstrapping analysis (with 5000 resamples) was performed to verify the mediating effects. The mediating effect was considered statistically significant if the 95% bootstrap confidence interval did not contain zero. The following criteria were used to appraise the model fit [22]: χ^2^/DF ≤ 5.00, comparative fit index (CFI) ≥ 0.90, the goodness of fit index (GFI), incremental fit index (IFI) ≥ 0.90, and standardized root mean square residual (SRMR) ≤ 0.08.

## Results

### Sample characteristics

A description of the characteristics of participants is presented in Table 1. The 460 adolescents with T1D were coming from 36 cities in 11 provinces in China. The mean age was 14.07 (*SD* = 3.95) years, ranging from 10 to 19 years. Half were female (54.1%), and 54.8% of the adolescents lived in rural areas. The age of diabetes onset and disease duration among participants was 9.89 (*SD* = 4.31) and 4.18 (*SD* = 3.46), respectively. The majority received insulin treatment using the insulin pen (72%), and the mean HbA1c was 8.14 ± 2.20. Concerning family status, more than half of the adolescents reported a family income over 5000 Yuan (54.7%) and lived in a single-child family (51.1%). Most parents of the adolescents received secondary or higher education (91.1%) and were employed (65%).

**Table 1:** Characteristics of participants (*N* = 460)

Variables Classification		N (%)/ Mean ± SD
Age, mean (SD), years		14.07 ± 3.95
Sex	Boy	211 (45.9)
	Girl	249 (54.1)
Residence	Urban area	208 (45.2)
	Rural area	252 (54.8)
Monthly family income, Yuan	< 5000	209 (45.3)
	≥ 5000	251 (54.7)
Single-child family	Yes	225 (48.9)
	No	235 (51.1)
Education level of parents	Junior high school or below	41 (8.9)
	high school	252 (54.8)
	College or above	167 (36.3)
Work condition of parents	Employed	299 (65.0)
	Unemployed	161 (35.0)
Age of diabetes onset, mean (SD), years		9.89 ± 4.31
Duration of diabetes, mean (SD), years		4.18 ± 3.46
Insulin therapy method	Insulin pen	331 (72.0)
	Insulin pump	129 (28.0)
Daily blood glucose monitoring	Yes	402 (87.4)
	No	58 (12.6)
HbA1c, mean (SD), %		8.14 ± 2.20

*Note* Data are represented in n (percentage) unless otherwise stated; SD, standard deviation

The results of the series of univariate logistic regression analyses showed that age of diabetes onset, insulin therapy method, age, gender, residence, education level and work condition of parents, family income, and family structure differed statistically significantly between adolescents in low resilience (DSTAR-Teen score ≤ 34) and high resilience group (DSTAR‐Teen score ≥ 46). These variables were used to perform multivariate analysis, and four variables were entered into the final stepwise linear regression model (Table 2). These variables were age of adolescents (*β* = 0.22, *P* < 0.001), family income (*β* = 0.14, *P* = 0.002), and education level of parents (primary or secondary education: *β* = 0.42, *P* < 0.001, primary or higher education: *β* = 0.26, *P* = 0.002). The multivariate analyses predicted 14.1% of the total variation in resilience.

**Table 2:** Regression-derived coefficients and significance of factors affecting resilience (*N* = 460)

	Adjusted R^2^	Standardised β	t	P
Education level of parents^†^	0.06	0.42	4.94	< 0.001
Age	0.11	0.22	4.94	< 0.001
Education level of parents^‡^	0.13	0.26	3.14	0.002
Monthly family income	0.14	0.14	3.06	0.002

*Note* For stepwise linear regression, included variables are age (years), gender, residence (rural or urban), monthly family income (< 5000 or ≥ 5000 Yuan), single-child family (no or yes), education level of parents^†^ ( primary or secondary education), education level of parents^‡^ (primary or higher education), work condition of parents (unemployed or employed), insulin therapy method (pen or pump), and age of diabetes onset (years)

### Correlations between family functioning, peer relationships, coping, and resilience

Table 3 presents the mean scores of study variables among adolescents with T1D, alongside the norms from normal adolescent populations in China. The mean scores for resilience, family functioning, peer support, peer stress, positive coping, and negative coping were 40.04 (*SD* = 9.21), 117.30 (*SD* = 20.11), 15.81 (*SD* = 6.10), 7.93 (*SD* = 3.35), 26.21 (*SD* = 8.24), and 6.86 (*SD* = 4.49), respectively. Resilience was positively associated with family functioning (*r* = 0.59, *P* < 0.001), peer support (*r* = 0.40, *P* < 0.001), positive coping (*r* = 0.57, *P* < 0.001), while negatively associated with peer stress (*r* = -0.34, *P* < 0.001). The association between resilience and negative coping was non-significant (*P* = 0.300). Moreover, family functioning was positively associated with peer support (*r* = 0.27, *P* < 0.001) and positive coping (*r* = 0.55, *P* < 0.001). Peer support was positively associated with positive coping (*r* = 0.33, *P* < 0.001). Peer stress was negatively associated with family functioning (*r* = -0.22, *P* < 0.001), peer support (*r* = -0.43, *P* < 0.001), and positive coping (*r* = -0.24, *P* < 0.001).

**Table 3:** Levels and associations among study variables (*N* = 460)

			Correlation Matrix					
	**Mean (SD)**	**Norms** ^a^	**1**	**2**	**3**	**4**	**5**	**6**
1. Family functioning	117.30 (20.11)	139.11	1					
2. Peer support	15.81 (6.10)	-	0.27^**^	1				
3. Peer stress	7.93 (3.35)	-	-0.22^**^	-0.43^**^	1			
4. Positive coping	26.21 (8.24)	33.19	0.55^**^	0.33^**^	-0.24^**^	1		
5. Negative coping	6.86 (4.49)	16.29	-0.01	0.02	0.16^**^	0.13^**^	1	
6. Resilience	40.04 (9.21)	-	0.59^**^	0.40^**^	-0.34^**^	0.57^**^	-0.05	1

*Note* ^**^Correlation is significant at the 0.01 level (2-tailed). ^a^ Normal adolescent population in China

### The ecological resilience model for adolescents with type 1 diabetes

Family functioning, peer support, positive coping, and resilience were modeled as second-order latent constructs with two dimensions. The results of CFA revealed acceptable factor loadings for family functioning dimensions (0.60 to 0.81) and peer support dimensions (0.69 to 0.86). Factor loadings for dimensions of positive coping (0.51 to 0.73) and resilience (0.65 to 0.96) were also acceptable. Moreover, the item factor loadings for the single dimension peer stress scale ranged from 0.62 to 0.84.

**Fig. 2:**
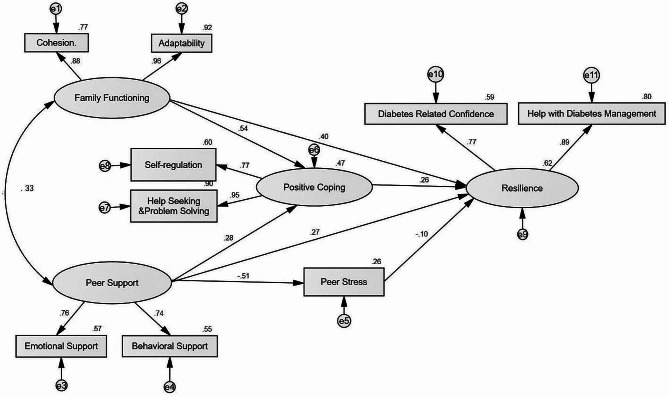
The ecological resilience model for adolescents with T1D

Negative coping was removed from the hypothesized model because the correlation coefficient *r* between negative coping and resilience was non-significant. The initial model indicated a acceptable model fit (χ^2^/DF = 4.276; CFI = 0.971, GFI = 0.961, IFI = 0.971, SRMR = 0.040). All paths of the model were statistically significant at *P* < 0.05, except the path from peer stress to positive coping (*P* = 0.646). The non-significant path was removed from the final model and the model fit was improved (χ^2^/DF = 4.073; CFI = 0.971, GFI = 0.964, IFI = 0.971, SRMR = 0.039). Compared to the initial model, the final model was further supported by a smaller AIC value(AIC _initial_ = 143.253 vs.AIC _final_ = 131.459). The coefficients for all paths are shown in Fig. 2.

Overall, family functioning, peer support, peer stress, and positive coping explained 62% of the variance in resilience. Standardized direct, indirect, and total path estimates are summarized in Table 4. Family functioning is the most important predicting variable of resilience (*β* = 0.40, *P* < 0.001), followed by peer support (*β* = 0.27, *P* < 0.001), positive coping (*β* = 0.26, *P* < 0.001), and peer stress (*β* = -0.10, *P* = 0.049). Positive coping was the mediator of the relationship between family functioning and resilience (*β* = 0.14, *P* < 0.001). Positive coping and peer stress co-mediated the association between peer support and resilience (*β* = 0.13, *P* < 0.001).

**Table 4:** Standardised direct, indirect, and total effects of variables in the ecological resilience model for adolescents with T1D (*N* = 460)

Endogenous Variables	Predicting Variables	Direct effect		Indirect effect		Total effect	
		*β*	*P*	*β*	*P*	*β*	*P*
Peer stress	Peer support	-0.51	< 0.001			-0.51	< 0.001
Positive coping	Family functioning	0.54	< 0.001			0.54	< 0.001
	Peer support	0.28	< 0.001			0.28	< 0.001
Resilience	Family functioning	0.40	< 0.001	0.14	< 0.001	0.54	0.001
	Peer support	0.27	< 0.001	0.13	< 0.001	0.39	< 0.001
	Peer stress	-0.10	0.049			-0.10	0.049
	Positive coping	0.26	< 0.001			0.26	< 0.001

## Discussion

In this study, the mean family functioning score among adolescents with T1D is 117.30, lower than the observed mean of 139.11 in a normal adolescent population in China [28]. Seiffge’s research also revealed that families of adolescents with T1D reported more structured, less cohesive, and less stimulating interactions compared to families of healthy adolescents [29]. Additionally, the ratio of positive to negative coping (26.21/6.86) was higher among adolescents with T1D than in the normal adolescent population in China (33.19/16.29) [30], indicating a stronger inclination among adolescents with T1D to employ positive coping strategies. Lastly, the mean resilience score among participants in this study (40.04) closely aligns with the resilience score of adolescents with T1D in the United States (45.40) [5].

The ecological resilience model constructed in this study suggested that family functioning was the most significant predictor of resilience among adolescents with T1D, followed by peer support, positive coping, and peer stress, explaining 62% of the variance in resilience. In addition, the stepwise linear regression revealed the three demographic factors of adolescents’ age, family income, and parents’ education level, accounting for 14% of the variance of resilience. These findings suggested that particular attention should be given to adolescents with younger age, lower family income, or parents with less education.

As hypothesized, family functioning was positively associated with adolescents’ resilience, which supported the previous findings [31, 32]. Moreover, we found that family functioning also indirectly influenced resilience through positive coping. Shao et al. also reported that coping style mediated the relationship between family functioning and resilience in adolescents whose parents with lung cancer [33].

The current study provides initial evidence that peer stress was negatively associated with resilience. Adolescents with T1D experience substantial peer stress because they need to conduct self-care behaviors throughout the day at school or during social events in the presence of others [34]. Fear of negative reactions from friends and schoolmates causes adolescents to conceal their T1D and withdraw from social outings, increasing feelings of isolation and stigma and subsequently impairing resilience [35]. In contrast, we found that peer support was positively associated with resilience in adolescents with T1D. This result coincided with previous studies in which a positive association between peer support and resilience was observed among youth who experienced disasters [36] or early left behind [37]. A qualitative study showed that emotional and self-management support from peers who also had T1D was important for adolescents in fighting the disease and obtaining resilience [38]. Moreover, we found that peer stress and positive coping mediated the association between peer support and resilience. A possible explanation may be that supportive relationships with peers may encourage adolescents to express their feelings and concerns and may thus reduce peer conflict. Chen et al. also proved the mediating roles of positive coping in the relationship between peer support and resilience among pregnant women [39].

In the final model, positive coping was directly associated with resilience among adolescents with T1D. Coping strategies play a fundamental role in managing developmental and disease-related stress [40]. As shown in other studies, pediatric patients with positive coping strategies (e.g., problem-solving, positive appraisal, and seeking support) had high levels of resilience [41]. Further, coping interventions have successfully improved resilience for school adolescents and patients with chronic disease [42]. Contrary to our hypothesis, the negative coping and resilience association was non-significant. Recent studies also found no correlation between defensive/evasive coping and resilience [41, 43]. According to the Social Ecology of Resilience Framework [8], the protective and risk factors interplay, where a balance of more protective factors than risk factors often lead to resilience.

Our findings have practical implications for healthcare providers caring for adolescents with T1D. First, strategies to enhance family functioning and peer support should be addressed simultaneously in the resilience promotion programs. For example, reduce parents’ psychological complaints and guide parents in discovering children’s strengths; facilitate regular dialogues between peers and adolescents with T1D regarding each other’s feelings and needs. Second, more attention should be given to promoting positive coping, such as problem-solving, environmental restructuring, and motivational interviewing, rather than reducing negative coping. Lastly, this study has provided a preliminary ecological model about resilience in adolescents with T1D. Healthcare providers should give increased attention to other vulnerable groups, such as children with rare diseases.

To our best knowledge, this is the first study to investigate the ecological factors associated with resilience among adolescents with T1D using a structural equation model. However, the current study is limited in several ways. First, the cross-sectional design hinders the definitive establishment of causality among the variables. More longitudinal work is needed to verify the relationships. Second, the participants were invited on a no-random and voluntary basis, which may lead to biased results, as volunteers generally have healthier psychological states. Third, the current study was conducted on Chinese adolescents with T1D, and the generalizability of the results to other populations might be interpreted with caution. Fourth, due to the limited time and energy of the adolescents, only family functioning and peer relationships within the microsystem were investigated. Future studies should consider assessing additional potential environmental ecological factors from the exosystems and macrosystem, such as policy and culture.

## Conclusion

The present study established an ecological resilience model for comprehensively understanding how resilience of adolescents with T1D is affected by family functioning, peer relationships, and individual coping style. The model fits well with the data and explains a large amount of the variance observed in resilience. More research is required to learn how to best enhance resilience in adolescents with T1D according to the model proposed in this paper.

## Data Availability

The datasets used and/or analysed during the current study are available from the corresponding author on reasonable request.

## Abbreviations

T1D: Type 1 Diabetes
HbA_1c_: glycosylated hemoglobin
DSTAR-Teen: Diabetes Strengths and Resilience Measure for adolescents
FACES II: Family Adaptability and Cohesion Evaluation Scales II
DSPSM: Diabetes-Specific Peer Support Measure
DSQY: Diabetes Stress Questionnaire for Youths
SCSQ: Simplified Coping Style Questionnaire
SD: Standard Deviations
SEM: Structural Equation Modeling
CFA: Confirmatory Factor Analysis
